# Considerations for Selecting Cognitive Endpoints and Psychological Patient-Reported Outcomes for Clinical Trials in Pediatric Patients With Sickle Cell Disease

**DOI:** 10.3389/fneur.2022.835823

**Published:** 2022-06-21

**Authors:** Anna M. Hood, Lori E. Crosby, Hanne Stotesbury, Melanie Kölbel, Fenella J. Kirkham

**Affiliations:** ^1^Division of Psychology and Mental Health, Manchester Centre for Health Psychology, University of Manchester, Manchester, United Kingdom; ^2^Division of Behavioral Medicine, Cincinnati Children's Hospital Medical Center, Cincinnati, OH, United States; ^3^Department of Pediatrics, University of Cincinnati College of Medicine, Cincinnati, OH, United States; ^4^James M. Anderson Center for Health Systems Excellence, Cincinnati Children's Hospital Medical Center, Cincinnati, OH, United States; ^5^Developmental Neurosciences Unit and Biomedical Research Centre, University College London Great Ormond Street Institute of Child Health, London, United Kingdom

**Keywords:** executive function, processing speed, depression, anxiety, intervention

## Abstract

Pediatric patients with sickle cell disease (SCD) experience a range of medical complications that result in significant morbidity and mortality. Recent advances in prophylactic and curative treatment approaches have highlighted the need for sensitive and clinically-meaningful trial endpoints. The detrimental effects of cognitive and psychological difficulties on social and economic mobility are well described. Although numerous reviews have assessed cognitive outcomes in other rare genetic disorders, SCD has not received the same focus. This review describes the cognitive (i.e., executive function and processing speed) and psychological domains (i.e., depression and anxiety) that are consistently associated with SCD pathology and, therefore, may be of particular interest as clinical trial endpoints. We then discuss corresponding well-validated and reliable cognitive tests and patient-reported outcomes (PROs) that may be appropriate for clinical trials given their robust psychometric properties, ease of administration, and previous use in the SCD population. Further, we provide a discussion of potential pitfalls and considerations to guide endpoint selection. In line with the move toward patient-centered medicine, we identify specific tests (e.g., NIH Toolbox Cognition Module, Wechsler Cancellation Test) and psychological PROs (e.g., PROMIS depression and anxiety scales) that are sensitive to SCD morbidity and have the potential to capture changes that are clinically meaningful in the context of patients' day to day lives. In particularly vulnerable cognitive domains, such as executive function, we highlight the advantages of composite over single-test scores within the context of trials. We also identify general (i.e., practice effects, disease heterogeneity) and SCD-specific considerations (i.e., genotype, treatment course, and disease course, including degree of neurologic, pain, and sleep morbidity) for trial measures. Executive function composites hold particular promise as trial endpoints that are clinically meaningful, amenable to change, relatively easy to collect, and can be incorporated into the routine care of patients with SCD in various settings and countries.

## Introduction

Sickle cell disease (SCD) is an umbrella term for a group of inherited disorders that affect the structure of hemoglobin and reduce the overall oxygen-carrying capacity of the blood (1). SCD affects ~100,000 individuals in the United States (US) and between 50,000 and 60,000 individuals in Europe (2), who are mainly immigrants or the descendants of individuals from endemic areas such as Sub-Saharan Africa (3, 4). For many years, chronic blood transfusion and hydroxycarbamide have been the primary therapeutic tools for SCD. Chronic blood transfusion remains the gold-standard treatment for stroke prevention (5). The US and European guidelines (6, 7) also highlight that hydroxycarbamide should be available for all pediatric (>9-months of age) SCD populations, and there is abundant evidence for laboratory and clinical efficacy (8, 9). Bone marrow and stem cell transplantations have long remained the only clinically available curative treatment options, but there are significant risks, and donors must be closely matched with recipients for optimal outcomes (10, 11).

**Figure 1:**
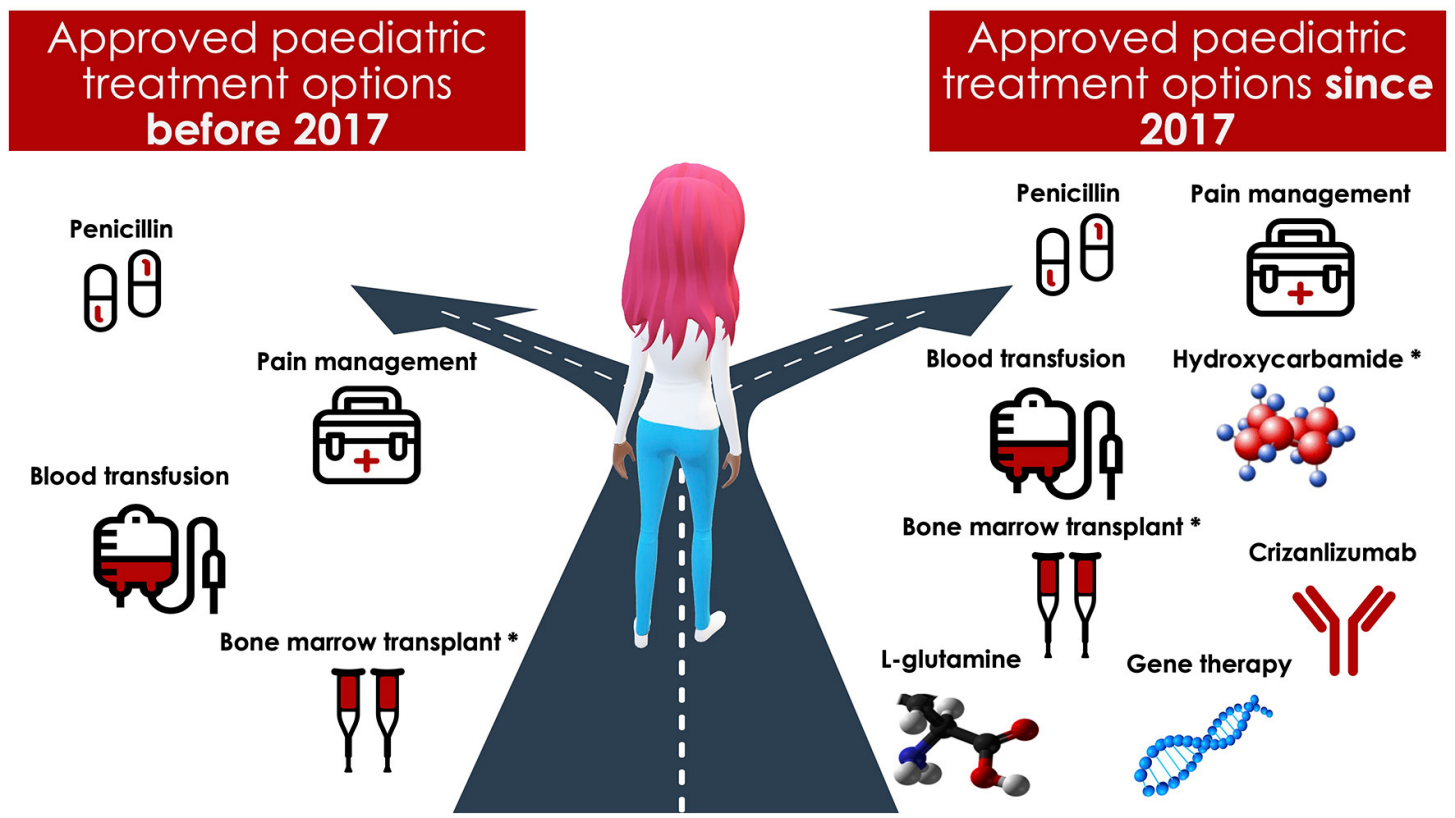
An overview of treatment options for pediatric patients with sickle cell disease. *Hydroxycarbamide was used off label for pediatric patients with sickle cell disease before 2017. Bone marrow transplantation requires a matched donor.

After years of stagnation, there has recently been an explosion in prophylactic and potentially curative treatment options for patients with SCD. The US Food and Drug Administration (FDA) has approved triple the number of new therapies within the past 4 years compared with the three decades prior. Among these treatments is the L-glutamine amino acid, Endari, which reduced oxidative stress and admissions for pain in a recent phase 3 trial (12). Others include Crizanlizumab, a humanized monoclonal antibody that binds to P-selectin, inhibiting adhesive interactions that may play a central role in pain episodes in SCD (13). Voxelotor (Oxbryta), a small molecule that binds to hemoglobin, inhibits hemoglobin polymerization and increases the hemoglobin's affinity for oxygen, was also recently approved (14). Although interest has also grown in curative therapies, including gene therapy (i.e., inserting genes to make healthy red blood cells) and gene editing (alteration of a selected DNA sequence in a living cell), these remain in the early stages of evaluation (15) (see Figure 1 for an overview of treatment options). Other innovative approaches currently under investigation in clinical trials in SCD include behavioral interventions (Clinical Trial No: NCT03150433) and Montelukast (Clinical Trial No: NCT04351698) (16) for comorbid sleep-disordered breathing (16).

Given the increase in novel therapeutic and curative approaches for the treatment of SCD, identifying sensitive and clinically meaningful endpoints for clinical trials is a pressing issue. Cognitive deficits (17, 18) have been identified frequently in patients with SCD, with more profound deficits observed in those with more severe neurologic injury (i.e., infarction) (19). Additionally, patients with SCD experience disproportionately high rates of psychological difficulties (i.e., depression and anxiety) (20, 21). Although more than 100 reports have documented the effects of 82 treatments on cognitive outcomes in patients with other rare genetic disorders (22), these areas have not received the same focus in SCD. Highlighting the knowledge gap, the American Society of Hematology (ASH) and the American Food and Drug Administration (FDA) recently partnered to develop consensus recommendations for clinical trial endpoints for patients with SCD (23). The ASH report included a summary of suggested cognitive tests and psychological patient-reported outcome measures (PROs) to use as endpoints. Our goal is to build upon these broad recommendations and discuss in-depth cognitive and psychological PROs that may be appropriate for use in clinical trials whilst discussing specific factors and potential pitfalls that must be carefully considered in selecting cognitive and psychological endpoints for trials.

Systemic SCD vascular pathology may simultaneously affect multiple end-organs with direct and indirect effects on the brain (24), and cognitive outcomes represent the final common pathway (25). Therefore, cognition may be well suited to assess the functional benefit of new therapeutic approaches to vascular end-organ disease. Further, better psychological functioning is associated with improved patient-reported and functional life outcomes, including quality of life (26, 27) and scholastic and employment gains (28). Cognitive tests and psychological PROs may also have several distinct advantages as endpoints for patients with SCD, including their ability to reliably capture meaningful cognitive impairment and psychological difficulties, their rigorous validation in the population, and their sensitivity to change.

Despite the significant advances that have resulted from randomized controlled trials across all chronic illness pediatric populations, the proportion of worldwide pediatric trials remains low at 9% (29). Similarly, although children bear 25% of the global chronic disease burden (30), few medicines are approved specifically for children, with rates of off-label prescribing estimated as high as 90%. Further, one study demonstrated that 38% of pediatric studies had yet to be completed for many drugs (e.g., anti-infective) authorized for adult use up to a decade ago (31). Given the evidence that early intervention may significantly reduce the risk of acute events (32) and that over 40% of patients with SCD are children (33), pediatric clinical trials are urgently needed in this population.

With the evidence for the effects of SCD on cognition and the proliferation of disease-modifying therapies in the last few years, including cognitive and psychological endpoints in clinical trials is a vital next step that may improve the knowledge-base around clinically meaningful outcomes in pediatric patients with SCD. Although cognitive dysfunction (17, 18, 34) and psychological difficulties (20) have been reported in pediatric patients with SCD for many years, there has been a focus on documenting *all* observed challenges. Relatively less attention has been paid to the specific domains with the most significant deficits, likely to be related to the pathophysiology and potentially preventable or even reversible, even though further focus in these areas could inform the development of targeted interventions. In view of childhood being a critical window for intervention, our paper will focus on the pediatric SCD population (0–18 years) and present a description of the cognitive and psychological domains in which patients frequently experience difficulties. Additionally, this paper will identify well-validated and reliable cognitive tests and psychological PROs that are capable of capturing changes beyond any practice effects that are clinically meaningful in the day to day lives of pediatric patients with SCD, and which may therefore hold promise as trial endpoints.

## Selecting Cognitive and Psychological Domains

Selecting appropriate cognitive tests and psychological PROs as endpoints in clinical trials is not trivial. Endpoints are the analyzed parameters (e.g., change from baseline to 12 weeks in standardized cognitive scores) and should be relatively easy to collect, proximal to the disease or treatment, medically significant, meaningful to patients, families and providers, and ideally be available for incorporation into routine care in a variety of settings and countries (35). Cognitive tests and psychological PROs fit all of these criteria, and patients with SCD and their advocates have called for their inclusion as critical endpoints for clinical trials assessing disease-modifying therapies (36).

Although cognitive function encompasses a variety of domains, including general intelligence, language, visual-spatial abilities, and memory, patients with SCD appear to experience particular difficulties in the domains of executive function (37–39), processing speed (40), and attention (41, 42). Psychological functioning covers an equally broad range of domains, comprising behavior, emotion, social skills, and overall mental health. In the SCD population, however, depression and anxiety symptoms appear to be the most common psychological challenges (20, 21). Below, we consider these cognitive domains and areas of psychological functioning in which difficulties have been most consistently reported in patients with SCD, and which therefore may be of particular interest as clinical trial endpoints.

### Intelligence Quotient

Significantly reduced intelligence quotients (IQ) are often observed in patients with SCD, and IQ is the most frequently reported indicator of general cognitive abilities (17, 18). However, although IQ provides a single composite with robust statistical properties, we do not recommend using IQ as an endpoint in clinical trials that include pediatric patients with SCD (see Table 1 for a detailed consideration).

**Table 1:** Intelligence quotient (IQ)—considerations for use as a cognitive endpoint in clinical trials of pediatric patients with sickle cell disease.

**Description**	**Reasons may not be appropriate for clinical trials**	**Reasons may be appropriate for clinical trials**
IQ is a total score derived from a set of standardized subtests (i.e., verbal and perceptual reasoning) designed to assess human intelligence. Each specific subtest (raw score) is compared to other children in the same age group (normative sample). Generally, an average IQ is 100 with a standard deviation of 15. Sixty-eight percent of population scores lie between 85 and 115.	IQ represents an aggregate or global capacity comprising a set of related but distinguishable abilities (43, 44). IQ is also not designed to measure the extent of cognitive impairment in single domains (43, 44). IQ enables a compensation model, i.e., a deficit in one subtest (e.g., Block Design) may be compensated by better performance in another, related, but distinct subtest (e.g., Vocabulary) (45). An IQ composite may obscure meaningful differences among subtest scores, conflating relative strengths and weaknesses (45). If no global IQ effect is observed investigators may accept the null hypothesis erroneously. Some subtests that make up the IQ are less sensitive to change during a clinical trial period, i.e., the Vocabulary and Similarities subtests rely on crystallized intelligence (accumulated knowledge based on experience) (46) that are unlikely to demonstrate an increase in IQ through a short-term intervention.	The Wechsler Abbreviated Scale of Intelligence Second Edition (WASI-II 2 or 4 subtest versions) takes 15–30 min to complete, respectively, can be given to pediatric patients aged as young as 6 years of age, and IQ along with verbal comprehension and perceptual reasoning indices can be obtained (47). Researchers may want to screen for global intellectual disabilities obtained as a baseline outcome rather than as a primary endpoint.
	There is limited evidence that interventions to improve IQ have sustained effects after they end (48).	
	IQ also only captures a subset of cognitive abilities pertinent to everyday functioning. Scores do not comprehensively reflect abilities in areas that are particularly vulnerable in pediatric patients with sickle cell disease.	Researchers may want to match IQ across treatment and placebo arms obtained as a baseline outcome rather than as a primary endpoint.
	Full-scale IQ requires administering a minimum of 10 subtests, which can take between 1 and 2 h depending on the participant's age and the need for breaks. This assessment length may be burdensome on both the administrator and the participant.	
	Collecting and interpreting IQ data as an endpoint in a clinical trial may be logistically difficult and prohibitively expensive.	
	Pediatric patients with sickle cell disease tend to score relatively lower on subtests (i.e., Vocabulary and Similarities) included in estimates or shorter version IQ tests. These tests does not include working memory and processing speed subtests; thus, estimates of IQ may be overestimated.	

### Executive Function

Moving beyond IQ, domain-specific tests of cognition are likely better able to capture changes in pediatric patients with SCD within the context of a clinical trial. Executive function is the domain that has received the most attention in both the SCD and broader literature. Debates remain as to the far transfer (e.g., to other skills) and length of benefits (e.g., >1 year) following interventions to improve executive function (49). The relative influence of socioeconomic status on performance is also an area of debate (50). Nevertheless, there is evidence that executive function is trainable to a certain degree and that more training leads to more significant gains (e.g., dosage effects) (51). The core executive functions comprise higher-level cognitive processes composed of three interrelated core skills: inhibitory control, working memory, and cognitive flexibility (52). Inhibitory control involves resisting the expression of an instinctive response and/or impulse to do something. Working memory involves holding information in mind while performing one or more mental operations. Cognitive flexibility is the mental ability to switch between concepts, flexibly adjust to changing demands, and look at something from a different perspective (53).

The dorsolateral prefrontal cortex is a region of the frontal lobes associated with executive function (54), and alterations in functional connectivity in this brain region have been observed in patients with SCD (55). There is evidence that deficits in switching and inhibition may be moderated by lesion type and location in patients with SCD, with one study indicating that children with frontal lesions showed the greatest impairments (56). Another study found a diminished event-related potential component difference between error and correct responses in SCD children with frontal lesions, indicating weaker response monitoring systems (57). Given that the prefrontal cortex also mediates social behavior (58), interventions that improve executive functions could, in theory, also indirectly support improved quality of life via improved social behavior, which may reciprocally enhance executive function. Support for this theory has been demonstrated through a 6-month executive function and social information intervention administered in the classroom (e.g., preschoolers without SCD) that showed improved inhibition, visual attention, and flexibility along with improved social processing skills (59).

Using executive function as a cognitive endpoint in a clinical trial in pediatric patients with SCD has several advantages. In the general population, executive function has demonstrated more predictive power than IQ, with working memory more predictive of scholastic success (60) and childhood inhibitory control revealed as more predictive of adult outcomes, including physical and mental health, criminal activity, and quality of life (61). Cognitive flexibility also predicts the ability to bounce back from and adapt to negative life events and everyday stressors (62). Specific to patients with SCD, there is considerable empirical evidence that executive dysfunction is related to sleep (63), persistent pain (64), chronic fatigue (65), abnormal blood velocities (66), cerebral blood flow (67), and quality of life (68–70), which are all often domains that are targeted in clinical trials of patients with SCD. Additionally, non-randomized studies have demonstrated that computerized working memory training programs (38, 39) and proximity to a blood transfusion (37) improve executive function. Taken together, these studies provide evidence that executive function is amenable to change in populations with SCD and that performance in this domain may serve as a clinically meaningful endpoint in future trials.

### Processing Speed

Processing speed refers to processing information that can be sensed, perceived, understood, and responded to rapidly (71) and has been identified as a core component of attention (e.g., sustained, selective, and focused). Slowed processing speed can limit cognitive function in other domains (e.g., how much information can be attended to or encoded) (72); however, although interrelated, processing speed has been shown to be separable from other cognitive processes (73). Importantly, processing speed is a sensitive and specific cognitive domain for pediatric patients with SCD (40). Several studies have found slower latency rather than poorer accuracy between patients with SCD and controls, which indicates that slowed processing speed may mediate impairments across other cognitive domains (74–77). Similar to executive function, processing speed has also been associated with SCD morbidity, including reduced arterial oxygen content and white matter integrity (40) and increased oxygen extraction fraction, a potential marker of ischemic risk (67).

A Phase 1 randomized controlled trial (RCT) has also demonstrated improved processing speed (Cancellation subtest of the WISC) in twelve patients with SCD randomized to 6 weeks of auto-adjusting positive airway pressure treatment (APAP) (78). Processing speed was also the primary endpoint for a larger, longer trial of APAP in children and adults with SCD (79), and along with executive function, is the primary endpoint of the planned trial to improve sleep-disordered breathing in young children with SCD (16). Given the current evidence that improved processing speed is related to improved functional outcomes and has demonstrated change following treatment in an RCT, it is a potentially sensitive and clinically meaningful endpoint in clinical trials for patients with SCD. Researchers could also consider including a test of processing speed as a measured outcome and then controlling for it in analyses assessing change in executive function endpoint to determine if it has explanatory power.

### Attention

Attention is a complex set of processes that allow individuals to select and concentrate on relevant stimuli. There have been relatively few studies specifically on attention in pediatric patients with SCD (17). However, prevalence rates of ADHD in children with SCD appear to be higher than the general pediatric population estimate of ~10% (80), with studies conducted in the US finding rates between 19 and 40% (81–83). In pediatric patients with SCD, a pilot RCT has also demonstrated the short-term efficacy of stimulant medication in improving attention compared with placebo (41). Given that we have less evidence about attention in pediatric patients with SCD, at this time, we suggest that it should not be considered as a trial endpoint, particularly as so many different tests and measures have been used to assess this domain in a relatively small number of studies (17). However, attention tests could be included in trials as measured outcomes to learn if deficits are as widespread and persistent as those found in the executive function and processing speed domains.

### Depression and Anxiety

Depression and anxiety are diagnosable disorders that cause a persistent feeling of sadness and loss of interest, or a feeling of unease, such as worry or fear, respectively. The prevalence of depression in the pediatric SCD population is unclear, with estimates between 4 and 46% (84). These estimates are mostly much higher than the general population of Non-Hispanic Black adolescents and young adults (7–9%) (85). The prevalence of anxiety disorders is lower for children (8–17%) but may still significantly impact quality of life (86–88). Behavioral depression and anxiety-related interventions (in-person, mobile-app, pharmacological) using cognitive-behavioral therapy (CBT) (89) have been successful for patients with SCD in lowering negative thinking (90, 91) and improving coping skills (92–94). Similar to measures of attention, however, additional evidence is needed before depression and anxiety PROs (symptomology and diagnostic) should be used as endpoints in clinical trials assessing disease-modifying therapies in pediatric patients with SCD. Instead, we recommend that depression and anxiety are measured as outcomes within the context of a clinical trial to determine the relationship between cognitive domains and the therapeutic of interest.

## Cognitive Test and Psychological PROs Selection for Clinical Trials

The first, though often overlooked, consideration when choosing an endpoint is assessing whether the normative data collected from the test reflects the country's broad demographic characteristics in which testing is conducted, including factors such as age, racialised identity, sex, and educational status. Choosing a test can be challenging when assessing the majority Black SCD population, particularly on the African continent, as most tests are normed in countries with majority White populations (e.g., the US and the United Kingdom) using census data to determine the number of children from racialised identities. Moreover, no test is culture-free and cognitive processes such as visual perception and spatial reasoning can develop in culturally-distinct ways (95). To overcome these challenges, clinical trials could measure the change in raw (i.e., the actual score generated on a test) rather than standardized (i.e., normative age-scaled) scores. Clinical trials in which multiple institutions across different countries collect data must also determine whether the endpoint is appropriate for all institutions, particularly if some are high vs. low resource institutions or in countries in the Global North or South.

Many of the standardized cognitive tests and psychological PROs recommended in Tables 1, 2 are available in languages other than English, most often Spanish. Conducting language translations (e.g., from the original language to the target language) when the primary language of the population of focus is not available requires bicultural translators to generate culturally-responsive translations that address the discrepancies and cultural ambiguities that occur with text translations (102). Investigators should recognize that language adaptation of commercially distributed tests is not always possible as publishers may not help to facilitate this process (103) and that translating a test from one language to another does not eliminate the need to consider cultural influences.

**Table 2:** Neuropsychological test batteries previously used in the pediatric sickle cell population.

**Battery**	**Cognitive domain**	**Tests**	**Test time**	**Ages**	**Languages**	**Norms**	**Cost/admin**	**Where to get**	**Training needed**	**Scoring**
NIH toolbox cognition module (96)	*Executive*									
	Flexibility Inhibition Working memory	DCCS Flanker List sorting	4 min 3 min 7 min	3–85 3–85 7–85	English; Spanish; Cebuano	Introduced in 2012 *N* = 4,859 representative of U.S. Census population [gender, racialised identity, ethnicity (i.e., Hispanic), SES]	$500 per year (up to 10 iPads)/1 iPad per participant	Download from iTunes. Psychologist to unlock module; healthmeasures.net	Online; in-person; qualified users—psychometrist, psychology graduate students, or psychologist	On iPad—raw, age-corrected SS download to iCloud or email
	*Processing Speed*	Pattern comparison	3 min	7–85						
NIH examiner (97)	*Executive*									
	Flexibility Inhibition Working memory Fluency Planning	Set shifting Flanker CPT Anti-saccades Errors Dot counting N back Phonemic Category Unstructured	5 min 5 min 14 min 5 min 7 min 5 min 2 min 2 min 2 min 6 min	3–90	English; Spanish	Normed 2006–2010 *N* = 1,113 from 9 sites in the U.S. (range of sex, racialised identity, ethnicity). Range of disorders including 117 patients with SCD	Free/record forms; Computer with PsychoPy Version; has alternate forms	Available to qualified users upon email request http://memory.ucsf.edu/resources/examiner.	Training videos; qualified users—psychometrist, psychology graduate students, or psychologist	R (Statistical Software) included on the distribution CD
D-KEFS (98)	*Executive*									
	Flexibility	Trail making	10 min	8–89	English; Dutch; Danish; Norwegian; Swedish	Normed in 2000 *N* = 1,750 representative of U.S. Census population [gender, racialised identity, ethnicity (i.e., Hispanic), SES]	$1,000 kit and scoring, additional cost record forms; Annual license fee plus $1.25 per subtest/uses Q interactive on 2 iPads	Pearson clinical is available to qualified users, e.g., clinical psychologists	Training supervised by licensed/ registered clinical psychologists	By hand; computerized scoring kit; q- interactive (reports can be generated online)
	Inhibition	Color interference	12 min							
	Initiation	Design fluency	10 min							
	Fluency	Verbal fluency	7 min							
	Planning	Tower	15 min							
NEPSY-II (99)	*Executive*									
	Flexibility	Animal sorting	10 min	7–16	English	Normed in 2006 *N* = 1,200 representative of U.S. Census population [gender, racialised identity, ethnicity (i.e., Hispanic), SES]	$1,000 kit and scoring, additional cost record forms; Annual license fee plus $1.25 per subtest/uses Q interactive on 2 iPads	Pearson Clinical is available to qualified users, e.g., clinical psychologists	Training supervised by licensed/registered clinical psychologists	By hand; computerized scoring kit; q-interactive (reports can be generated online)
	Inhibition	Inhibition	11 min	5–16						
	Fluency	Design fluency	4 min	5–12						
	*Attention*	Auditory attention and response set	11 min	5–16						
Wechsler tests (WPPSI, WISC, WASI) (100)	*Processing Speed*	Coding	5 min	6–90	21 languages including English; Spanish; French; German Arabic; Chinese	Ns = 2,000–2,500 Representative of U.S. and U.K. based on census data (gender, racialised identity, ethnicity, SES). Separate norms available for other translations	$1,400 kit and scoring, $60 per year for scored reports; Annual license fee plus $1.25 per subtest using Q interactive on 2 iPads	Pearson Clinical is available to qualified users, e.g., clinical psychologists	Training supervised by licensed/ registered clinical psychologists	By hand; computerized scoring Q global; q- interactive (reports can be generated online)
		Animal Coding	5 min	4–7						
		Symbol Search	5 min	6–90						
		Bug Search	5 min	4–7						
		Cancellation	5 min	4–90						
Continuous performance test (101)	*Attention*	Kiddie CPT CPT	8 min 14 min	4–7 8–90	English	*N* = 1,400 representative of U.S. Census population [gender, racialised identity, ethnicity (i.e., Hispanic), SES]	$2,900 for USB with unlimited use of both tests	Pearson Clinics al available to qualified users, e.g., clinical psychologists	Training supervised by licensed/registered clinical psychologists	Computerized scoring on USB (reports are generated)

*SCD, sickle cell disease; Admin, Administration; DCCS, Dimensional Change Card Sort; Flanker, Flanker Inhibitory Control and Attention; SES, socioeconomic status; SS, standard scores; CPT, Continuous Performance Test; D-KEFS, Delis-Kaplan Executive Function System™; WPPSI, Wechsler Preschool & Primary Scale of Intelligence; WISC, Wechsler Intelligence Scale for Children; WAIS, Wechsler Adult Intelligence Scale*.

Clinical trials of pediatric patients with SCD can follow established guidelines for other conditions in which patients experience cognitive impairment (e.g., traumatic brain injury, Neurofibromatosis Type 1). For example, FDA guidance for drug trials of patients with a traumatic brain injury requires documenting cognitive tests before trial initiation, choosing tests and PROs guided by conceptual models, and providing adequate justification of the outcome measure(s) (104). When choosing tests for pediatric patients with SCD, it is essential to consider the pathways by which novel therapies improve SCD-related pathophysiology and how these may relate to the neurophysiological processes that support brain function. For example, drugs such as Voxelotor, which reversibly binds to hemoglobin and prevents HbS polymerization by increasing the hemoglobin's affinity for oxygen, could likely improve executive function by increasing hemoglobin concentration, reducing compensatory hemodynamic stress (14). The current randomized trial of Voxelotor, HOPE Kids 2, with a primary endpoint of change in transcranial Doppler velocities in children aged 2–16 years with SCD conditional velocities, includes tests of executive function and processing speed.

Future trials must consider the extent to which any observed changes are statistically significant and the extent to which they are clinically meaningful or functionally significant. For example, a cognitive test with good reliability may show a statistically significant 1 to 2-point change. Practically, however, this might not mean that the changes translate into functionally relevant benefits (e.g., scholastic, employment) within the context of patients' daily lives. Therefore, an effect size that would demonstrate meaningful change should be determined before study initiation. Moreover, researchers can calculate the minimal clinically important difference (MCID). MCID is the smallest difference in the cognitive tests and psychological PROs used as outcomes and endpoints that patients perceive as beneficial or harmful, i.e., what is actually important to patients (105). Linking the magnitude of change to clinical trial efficacy and effectiveness reflects the intention to find a clinically important treatment effect.

MCID can be calculated through the (1) the anchor-based method, i.e., by anchoring change on the PROMIS, a numerical scale, to a categorical response (e.g., a lot better), (2) by consensus (e.g., Delphi) methods, i.e., convening an expert panel to provide independent assessments of MCID, and (3) the distribution-based method, i.e., using the distribution of the outcome or endpoint scores, particularly the variation between patients. However, this method does not center on the patient (106). For the psychological PROs, previous research in adult samples has demonstrated a 3–4 point change on Patient-Reported Outcomes Measurement Information System (PROMIS) anxiety and depression (107) or a 5-point change on the Patient Health Questionnaire (PHQ-9) (96) are considered as MCID. MCID has been utilized less for cognitive tests, but a recent study found that for older adults assessing raw scores and completion times, MCID improvement over 1-year following an exercise program was 3–5 symbols for the Digit Symbol Substitution Test (a measure of processing speed) and −11.5 to −26.0 s for the Stroop (a measure of inhibition) (97).

There are several reliable cognitive test batteries (see Table 2) and psychological PROs (see Table 3) that meet the criteria defined above and that have been used in pediatric SCD populations (17). These tests provide measures that may therefore be good candidates for endpoints in clinical trials. To aid decision making, Tables 2, 3 include details on the normative data used for scoring (most often based on US Census data), the cost and where to obtain tests, along with the training necessary to administer and interpret results. Researchers will also have to determine whether to obtain child-report and/or caregiver-proxy reports for psychological PROs. This decision will often be reached by considering the child's age, weighing the burden to families, the complexity of having multiple reporters, and the additional cost of gathering and analyzing data. Child and caregiver proxy reports should not be aggregated as the poor reliability (i.e., low correlations) between child and caregiver ratings represent different perspectives (98).

**Table 3:** Psychological patient-reported outcomes frequently used in the pediatric sickle cell population.

**Measures**	**Psychological domain**	**Test time**	**Ages**	**Languages**	**Norms**	**Cost/admin**	**Where to get**	**Training needed**	**Scoring**
PROMIS (self-report and caregiver-proxy report) (108)	Depression Anxiety	5 min 5 min	5–90 5–90	English; Spanish; Other language translations on request	Ongoing validation; a range of disorders including patients with SCD	Free; on paper; computer; with an app $750 fee for other language translations (can request a waiver)	NIH Toolbox app; PROMIS app; REDCap; EPIC; OBERD; healthmeasures.net	Can train research staff, students quickly. Interpretation of scores from a psychologist	By hand; on iPad, download and score using statistical software
Beck depression inventory-II (BDI – II) (109)	Depression	5 min	13–80	English; Spanish;	*N* = 500 adult psychiatric outpatients in the US and a student sample of 120 college students in Canada as the control group	$40 per year for a single scoring subscription; $100 for starter kit; $60 for 25 paper record forms	Pearson clinical-available to qualified users, e.g., clinical psychologists	Can train research staff, students quickly. Interpretation of scores from a psychologist	By hand; on iPad, download and score using statistical software
Patient Health Questionnaire-−9 (PHQ-9) Adolescent version available (110)	Depression	2–5 mins	11–17 18 and over	40 languages including English; Spanish; Arabic; Chinese	*N* = 6,000 patients (3,000 from general internal medicine and family practice clinics *N* = 3,000 from obstetrics-gynecology clinics in the US	Freely available	Download online	Can train research staff, students quickly. Interpretation of scores from a psychologist	By hand; on iPad, download and score using statistical software
State-trait anxiety inventory (111)	Anxiety	10 min	15 and over	40 languages including English; Spanish; Arabic; Chinese	Large US sample of college and high school students Small groups of psychiatric, general and medical patients, and young prisoners	$100 Adult complete kit—manual with scoring key and 25 record forms	Mind Garden- available to qualified users, e.g., clinical psychologists	Can train research staff, students quickly. Interpretation of scores from a psychologist	By hand; on iPad, download and score using statistical software
Generalized Anxiety Disorder – 7 (GAD – 7) (112)	Anxiety	2–5 min	13 and over	40 languages including English; Spanish; Arabic; Chinese	*N* = 2,740 primary care patients *N*= 5,030 general population in Germany reported sex and other demographic characteristics, but not racialised identity or ethnicity	Freely available	Download online	Can train research staff, students quickly. Interpretation of scores from a psychologist	By hand; on iPad, download and score using statistical software
Child behavior checklist (CBCL) (113)	Emotional and behavioral problems	10 min	6–18	English; French; Translation into 60 languages	*N* = 2,300 children assessed at 42 mental health agencies in the US (this is used in the scoring software). The Multicultural Family Assessment Module on the Progress and Outcomes App provides norms from 50 other countries	$500 for the computerized starter kit	ASEBA—available to qualified users, e.g., clinical psychologists	Can train research staff, students quickly. Interpretation of scores from a psychologist	By hand; on iPad, download and score using statistical software

*SCD, sickle cell disease; Admin, Administration; PROMIS, Patient-Reported Outcomes Measurement Information System; EPIC, Electronic Portfolio of International Credentials; ASEBA, The Achenbach System of Empirically Based Assessment*.

## Cognitive Test and Psychological PROs Administration

**Figure 2:**
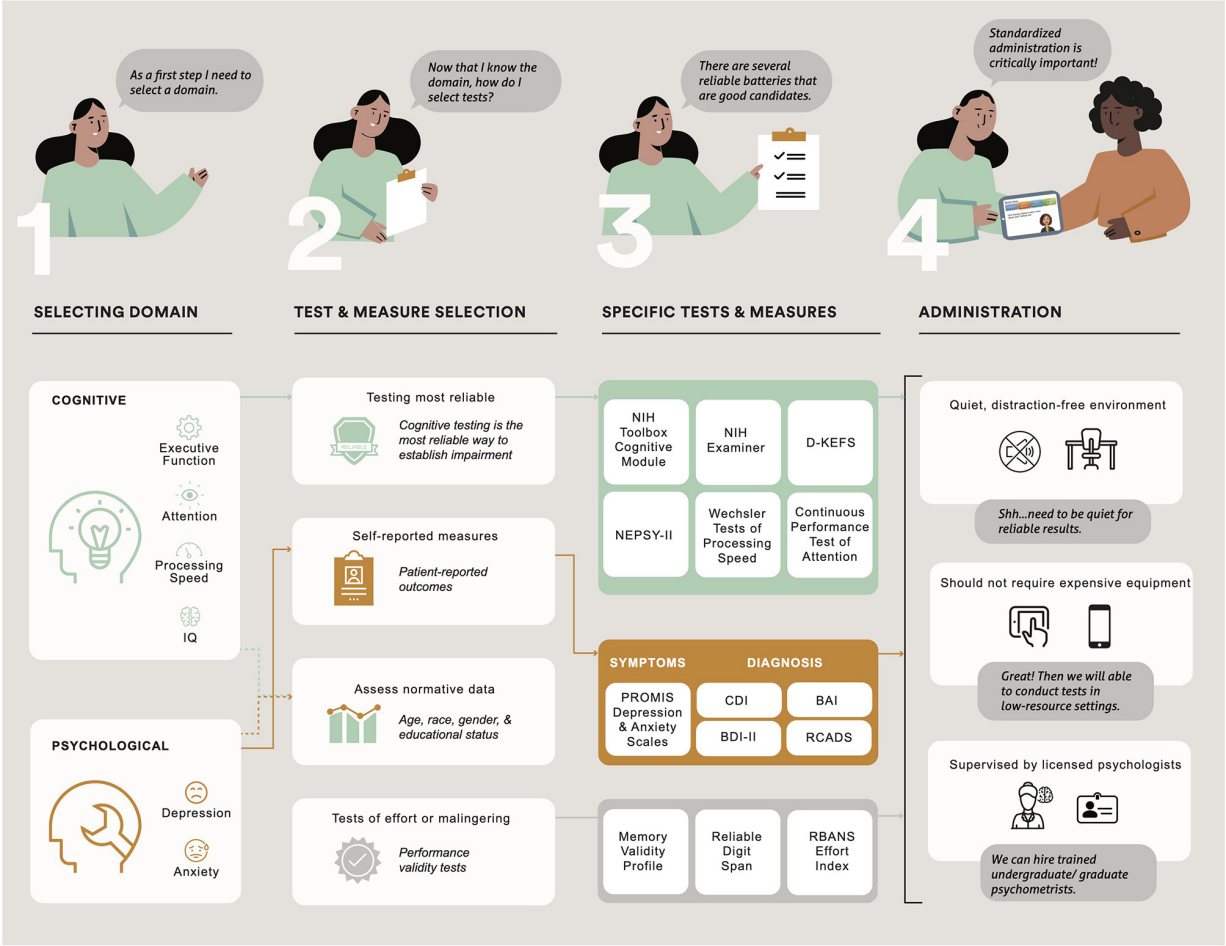
Description of the process in choosing cognitive domains and psychological function and administering cognitive tests and psychological patient-reported outcomes in clinical trials of pediatric patients with sickle cell disease. NIH, National Institutes of Health; D-KEFS, Delis Kaplan Executive Function System; NEPSY-II, A Developmental NEuroPSYchological Assessment; PROMIS, Patient Reported Outcome Measurement Information System; CDI, Children's Depression Inventory; BAI, Beck Anxiety Inventory, BDI, Beck Depression Inventory-II; RCADS, Revised Children's Anxiety and Depression Scale.

Standardized test administration is critically important so that scores obtained do not over or underestimate actual ability (99). All participants need a quiet, distraction-free environment, along with a precise reading of instructions and the provision of necessary tools or stimuli. Generally, psychometrists or graduate-level students with specialized training administer and score tests according to manual instructions. Psychologists qualified to interpret scores should directly and closely supervise (100). Cognitive testing and psychological assessment should optimally occur in locations and at times that reduce the burden on participants and are consistent across trial visits. When choosing tests for clinical trials in low-resource settings, investigators should consider tests that do not require time-consuming adaptations, are inexpensive enough to be administered as a part of usual care, and do not need expensive equipment (see Figure 2).

Many standardized tests now have the option for administration via an iPad (see Table 2). Computer-aided scoring and interpretation are also available for these instruments. These adaptations (e.g., stopwatch, audio recorder) make it much easier to test at multiple locations (e.g., SCD clinic), easing the burden on patients and their families who likely have many routine medical appointments to attend. Additionally, computer-based batteries appear to reduce administration errors and increase efficiency in testing (101). Disadvantages associated with iPad administration of some tests include the need to configure Wi-Fi to the test battery and the need for a blue-tooth connection for between-device communication during administration. There are several options for completing psychological PROs, including paper and pencil, phone, or online (e.g., REDCap or Qualtrics surveys).

The use of digital and remote assessment of psychological PROs in clinical trials has accelerated since the start of the COVID-19 pandemic (108). Although remote assessment may improve reliability and reduce variability if continuous or multiple data points are gathered, may be cost-effective, and appear more inclusive as they reduce transportation burden, there are disadvantages that researchers in clinical trials of pediatric patients with SCD need to scrutinize before making their decision. Remote assessment of psychological PROs may reduce control, require participants to have Wi-Fi connections, and personal computers, tablets or mobile phones, which may not be available to all families. Providing in-person (conducted during usual care appointments) and online options to complete psychological PROs is one way to lessen digital exclusion for patients and families (109).

### Practice Effects

Practice effects refer to improvements in performance due to increased familiarity with and exposure to test materials, differing test-taking strategies, and less anxiety in the test-taking environment. If not considered, such effects may be difficult to disentangle from effects related to the intervention of interest. Neuropsychologists generally schedule cognitive testing 1–2 years apart in clinical practice. Serial cognitive testing and psychological assessment occur more frequently in clinical trials, making practice effects a genuine concern. If not correctly integrated into the interpretation of results, practice effects can easily lead to false conclusions. Despite this, these effects are often underappreciated (100). Timed tests, psychomotor processing tests, and novel tests are more susceptible to practice effects (110). Unfortunately, these are the tests that patients with SCD generally find the most challenging (37). Additionally, practice effects tend to occur more often in younger vs. older participants (111). It is also possible that practice effects may differ between those with SCD and controls with disease severity (e.g., SCD genotype, pain episodes, sleep difficulties, brain abnormalities) in the pediatric SCD population serving to modify results. However, this possibility has not yet been empirically tested. When comprising their samples, researchers should weigh the potential for practice effects (e.g., large age range, choice of tests) when choosing appropriate endpoints for the clinical trial.

There are approaches to reduce practice effects in clinical trials (112). First, massed practice in a pre-baseline period (e.g., showing all participants the test materials, providing instructions, completing test practice items) may reduce the effects of novelty and any anxiety related to the test-taking environment. The precise number of pre-baseline assessments necessary to achieve habituation (i.e., for cognitive performance to stabilize) has not been established, but previous research in non-SCD populations has indicated that 1 to 2 practice trials before baseline can result in habituation (113). Using massed practice would have to be considered carefully, however, given the additional cost and time burden. Second, if it is possible to counterbalance the administration of tests (e.g., systematic variation of the order of conditions), it can help to reduce practice effects as well as the influence of other nuisance variables. Third, computerized adaptive testing (e.g., tests that adapt to the participant's ability) can reduce practice effects (114). Fourth, using tests with multiple similar items or alternate forms of tests at baseline and post-intervention can minimize item exposure. Specific to patients with SCD, researchers could consider using the Wechsler Cancellation subtest when assessing processing speed as previous research demonstrates that this test appears more resistant to practice effects in this population (78). Each approach has strengths so that the final decision will depend on economic and pragmatic considerations (112).

## Other Considerations

### Age

A clinical trial of pediatric patients with SCD will need to consider the influence of normal growth and development on cognitive processes. For instance, children between the ages of 3–5 years have more difficulty with tests of executive function, and different executive functions mature at different rates (53). Attentional control emerges first in infancy and then develops quickly in early childhood. Cognitive flexibility and information processing take longer to develop and have a critical period between 7 and 9 years. They are relatively mature by 12 years of age. After a transitional period that begins in adolescence, “executive control” is thought to emerge (115). These developmental changes make assessing executive function in a pediatric population more challenging. For example, if a child is 7 years old at baseline and 9 years old at the end of a clinical trial, any improvements in executive function may be due to expected developmental growth rather than the disease-modifying therapy or intervention under consideration. As treatment effects are typically averaged across a sample, further challenges may arise if the sample includes children at very different developmental stages. In the example above, scores in those over the age of 12 may be expected to remain relatively unchanged. A specific examination of scores and/or a well-matched control sample (ethnicity, age, socioeconomic status) can help to determine if the change is related to age or the intervention.

Researchers may choose to analyze both raw and standardized scores. Raw scores might best fit the data (116) but are also susceptible to regression to the mean (the tendency for a person's score to move toward the population mean test score with retesting) and practice effects (117). Although changes in age-corrected standard scores are likely more understandable to the audience, their interpretation can be misleading. For example, a negative change in standard scores can either represent an actual decline in raw scores or a failure for raw scores to improve at the expected rate for age, with no change in raw scores or performance. In the context of a clinical trial, researchers may need to consider standardizing post-treatment scores using baseline age or assessing patients across a narrower age range (e.g., 12–16 rather than 6–16 years), examining developmental trajectories for the particular test.

### Genotype and Treatment Course

Several SCD-specific factors need to be considered when using cognitive tests as endpoints in clinical trials. Foremost, although most clinical trials target pediatric patients with the HbSS genotype, they also include other genotypes (e.g., HbSC, HbSβ0 thalassemia, HbSβ+ thalassemia). The patients with the HbSS genotype often experience the most clinical severity and consequently have more cognitive challenges than other patients with SCD (17). It is unclear if these patients also have more psychological challenges. Moreover, if patients continue to receive treatments (e.g., chronic blood transfusion, HU) as part of standard care during the clinical trial, cognitive endpoints may differ related to treatment type (118) or when the patient received treatment in relation to cognitive testing (37). As such, researchers may need to account for genotype and treatment course and decide whether to minimize for these factors to ensure balance across trial arms, divide samples into subgroups, or control for these factors in their analyses.

### Neurologic Complications

Previous research has shown that compared to controls, children with SCD with and without overt neurologic complications have a higher burden of white matter hyperintensities (119), reduced cortical and subcortical gray matter volume (120), and widespread reductions in the microstructural integrity of white matter (40, 121). Past work also demonstrates that pediatric patients with SCD who have experienced overt stroke consistently have poorer cognitive outcomes than other patients with SCD and non-SCD controls (19). The literature is more mixed concerning cognitive differences between patients with SCD and silent cerebral infarction (SCI) and those with “normal-appearing MRI.” Although older studies have tended to find a detriment in cognitive performance in patients with SCI (17, 122), more recent studies have found few, if any, differences (40, 123–125). Discrepancies in the literature may relate not only to differences in the precise definition of SCI, sample characteristics, and cognitive domains studied but also to advances in MRI technology, with higher resolution scans identifying a greater number of patients (and controls) with SCI (126). With higher resolution techniques, other characteristics such as lesion volume may provide a more sensitive metric.

Given these challenges, if mixed neurologic groups are included in a clinical trial, data from patients with extensive structural tissue injury could be analyzed separately to determine if they differ significantly from the larger sample. Alternatively, investigators may control or minimize for neurologic status in analyses. Deciding whether to analyse data from patients with and without SCI will require deeper investigation of findings and, ideally, reporting why data have been grouped (e.g., scores were very similar) or not grouped (e.g., clinically meaningful differences). However, researchers conducting analyses in subgroups of pediatric patients with SCD (e.g., stroke vs. SCI vs. no infarct on MRI) will have to balance the additional knowledge gained with reduced statistical power, as sample sizes will be made smaller by subgrouping. Another possible route includes using block randomization so that there is more than one patient group with equal numbers of participants with overt stroke and SCI (presence/volume) assigned to each group.

### Pain

Although pain causes significant morbidity for those living with SCD, our understanding of the impact on cognition remains limited. Nevertheless, the few studies conducted show that persistent pain and executive dysfunction are significantly related (64). Coordinating cognitive testing only when patients are not experiencing pain can be a goal. However, this may generally not be achievable given that many patients with SCD experience persistent pain (127, 128). One potential option is to use pain-related PROs to determine potential pain interference and intensity and their relation to cognitive endpoints. Pain experience/frequency may then be included as a control variable in analyses (129). Minimization for baseline pain is another alternative.

### Sleep

Previous research indicates that pediatric patients with SCD experience sleep-disordered breathing (130), including obstructive sleep apnoea (131), along with a high prevalence of sleep-onset insomnia (132). Other sleep disturbances (133), including nocturnal enuresis (134) and leg movement (135), occur in about one-third of patients. There is evidence that sleep may impact cognitive performance (136). In clinical trials of patients with SCD, including a measure of sleepiness as an outcome would therefore be useful (137). Researchers should also consider whether they will use a predefined “sleepiness” cut-off score to determine if cognitive testing can be conducted. Researchers might consider choosing the most suitable time of day for each patient to be tested (138). Utilizing these measures might increase the time burden and require additional coordination with families, as the timing of testing would need to be coordinated with greater precision and ought ideally to be matched at both baseline and study exit. Similar to our suggestions related to pain, accounting for sleepiness in statistical analyses is another way to control for this factor.

## Cognitive Composite Endpoints

Combining test scores or item responses from relevant subdomains into a composite score creates a single endpoint with several advantages for clinical trials in pediatric patients with SCD. Multiple endpoints may appear to improve the explanatory power and provide additional specificity; however, they also require assumptions about the magnitude of the treatment effect across outcome domains and increase the possibility of *post-hoc* “cherry-picking” of significant results (104). Composite endpoints may simplify decision making around selecting a specific primary outcome from multiple plausible tests or measures (139). The FDA requires a single outcome, defined *a priori*, to license new drugs (140). In a rare disorder like SCD, the additional statistical power afforded by a single endpoint cannot be discounted. The easiest and most widely used method to create a composite is to place the scores on a common metric (e.g., z, T, scaled score, or other standard scores) and then average them (141). An executive composite created using this method differentiated between children with SCD receiving chronic transfusions, HU, and demographically-matched controls (37). As this method does not assess inter-correlations between tests or the factor structure, it can provide a summary score of overall cognitive performance in a particular domain (141).

An alternative approach is to use confirmatory factor analysis to model latent ability based on a set of scores. The latent ability model captures variance across tests or measures and accounts for covariance attributable to method effects or theoretical similarities. For example, if two tests both measure cognition with precision, but one test measures higher-order functioning and the other lower-order functioning, the latent ability model can measure the underlying trait across the full range of ability. An additional advantage is that this methodology can control for and quantify differences in performance based on demographics (e.g., age, sex at birth). Once computed, a latent ability composite score (mean = 0, standard deviation = 1) is available for each participant in the study (104). Latent ability composite scores have been created for Alzheimer's disease studies (142) but not yet for adult or pediatric SCD studies.

We advocate for a single cognitive endpoint in clinical trials of patients with SCD, like an executive function composite. Notably, a domain-specific composite is not the same as an IQ, which is an aggregate or global index that reflects performance across a wide variety of cognitive domains. There are inherent challenges in developing composites as tests in the same domain are not identical and may capture distinct abilities. Batteries of executive function tasks balance both unity and diversity, but the three target executive functions (e.g., cognitive flexibility, inhibition, working memory) do tap into an underlying common ability (52, 143), and there is evidence of dysfunction in all three areas of executive function in SCD (17). The NIH EXAMINER already provides an executive composite score that has been shown to differentiate between groups of children with SCD with and without silent infarction and stroke (144). However, most neuropsychological batteries with demonstrated sensitivity to executive dysfunction in SCD do not provide a specific executive function composite, so either the averaging or latent approach will need to be considered.

## Conclusion

This paper identifies specific tests (e.g., NIH Toolbox Cognition Module, Wechsler Cancellation Test) and psychological PROs (e.g., PROMIS depression and anxiety scales) that can potentially capture clinically changes meaningful in the context of patients' day to day lives. For cognition, executive function and processing speed are the domains in which pediatric patients with SCD have the most difficulty. There is preliminary evidence that executive function composite scores are amenable to disease-modifying therapies (37–39); therefore, they hold particular promise as endpoints for future clinical trials, with batteries such as the NIH Toolbox and the NIH Examiner providing valid and reliable measures with demonstrated sensitivity to change. A significant proportion of pediatric patients with SCD have widespread brain abnormalities, including in the prefrontal cortex of the brain (145), executive function encompasses those cognitive processes that underlie goal-directed behavior mediated by activity within the prefrontal cortex, and executive function has been demonstrated to be related to cerebral hemodynamic parameters (67). Therefore, it is unsurprising that executive function is a plausible candidate endpoint for a clinical trial.

Cognitive tests and psychological PROs do not come without limitations and specific considerations for the SCD population, but thoughtful adaptations to study design and statistical analyses may help address potential challenges. Although not intended as an exhaustive list, this review provides an overview of recommended tests and PROs that are relatively easy to collect, associated with SCD morbidity, meaningful to patients and families, and can be incorporated into routine care in various settings and countries.

## Author Contributions

AH conceptualized the paper, completed the review, and drafted the manuscript. HS and MK contributed to the review and provided feedback on the manuscript. LC and FK conceptualized the paper, contributed to the review, and provided feedback on the manuscript. All authors contributed to the article and approved the submitted version.

## Funding

AH was supported in part by a grant from the National Heart, Lung, and Blood Institute (1F32HL143915). LC was funded through a Patient-Centered Outcomes Research Institute (PCORI) Award (CDR-1609-36055).

## Conflict of Interest

The authors declare that the research was conducted in the absence of any commercial or financial relationships that could be construed as a potential conflict of interest.

## Publisher's Note

All claims expressed in this article are solely those of the authors and do not necessarily represent those of their affiliated organizations, or those of the publisher, the editors and the reviewers. Any product that may be evaluated in this article, or claim that may be made by its manufacturer, is not guaranteed or endorsed by the publisher.
